# A Two-Step Method for Impact Source Localization in Operational Water Pipelines Using Distributed Acoustic Sensing

**DOI:** 10.3390/s25154859

**Published:** 2025-08-07

**Authors:** Haonan Wei, Yi Liu, Zejia Hao

**Affiliations:** 1 State Key Laboratory of Water Cycle and Water Security, China Institute of Water Resources and Hydropower Research, Beijing 100038, China; liuyi@iwhr.com; 2 China South-to-North Water Diversion Middle Route Corporation Limited, Beijing 100038, China; haozejia@126.com

**Keywords:** pipeline monitoring, distributed acoustic sensing, source location, signal extraction, arrival time picking

## Abstract

Distributed acoustic sensing shows great potential for pipeline monitoring. However, internally deployed and unfixed sensing cables are highly susceptible to disturbances from water flow noise, severely challenging impact source localization. This study proposes a novel two-step method to address this. The first step employs Variational Mode Decomposition (VMD) combined with Short-Time Energy Entropy (STEE) for the adaptive extraction of impact signal from noisy data. STEE is introduced as a stable metric to quantify signal impulsiveness and guides the selection of the relevant intrinsic mode function. The second step utilizes the Pruned Exact Linear Time (PELT) algorithm for accurate signal segmentation, followed by an unsupervised learning method combining Dynamic Time Warping (DTW) and clustering to identify the impact segment and precisely pick the arrival time based on shape similarity, overcoming the limitations of traditional pickers under conditions of complex noise. Field tests on an operational water pipeline validated the method, demonstrating the consistent localization of manual impacts with standard deviations typically between 1.4 m and 2.0 m, proving its efficacy under realistic noisy conditions. This approach offers a reliable framework for pipeline safety assessments under operational conditions.

## 1. Introduction

Pipelines serve as critical infrastructure for transporting water resources over long distances. However, these structures are susceptible to various threats throughout their whole length, including aging, corrosion, ground movement, and third-party intrusion (TPI), such as accidental impacts from construction activities [1,2]. For specific types, like prestressed concrete cylinder pipes (PCCPs), which are often used in large-scale water transport systems, failures like wire breaks also pose significant risks [3]. Pipeline ruptures not only result in the waste of water resources but also lead to the loosening of surrounding soil and reduced foundation-bearing capacity, thereby affecting both residential and industrial activities. Unlike gradual degradation processes such as leakage, sudden anomalous events like impacts are often unpredictable and can lead to immediate and catastrophic failure, necessitating effective real-time monitoring [4]. Oil and gas pipelines face even greater challenges from TPI. According to statistics from the China Gas Association, nearly 60% of pipeline incidents are caused by TPI [5]. Therefore, protecting structural integrity against impact damage caused by construction activities is crucial for preventing environmental and social problems.

Traditionally, pipeline intrusion monitoring relied on point-type sensors, such as accelerometers [6], hydrophones [7], microphones [8], and Fiber Bragg Grating [9]. Chen et al. [10] utilized a piezoceramic transducer array to capture impact-induced stress waves and estimated the time of arrival using instant phase analysis, enabling simultaneous axial and circumferential localization of impact sources on pipeline. The sensors were installed at certain intervals along the pipeline to detect the surrounding vibration. However, due to the attenuation of vibration waves during propagation, these sensors need to be densely deployed to ensure an adequate signal-to-noise ratio in practical applications, resulting in high installation costs.

Recently, distributed acoustic sensing (DAS) technology has offered advantages such as long continuous monitoring, a high spatial sampling density, and low underwater protection requirements [11,12]. DAS technology is based on the detection of the phase change in the lightwave within the optical fiber, which is laid along the pipeline, capturing environmental vibrations and transmit signals [13]. As a result, the vibration signals detected by DAS are not affected by attenuation, unlike those from point-type sensors. By monitoring the vibration signals recorded by the optical cable deployed along the pipeline, vibration events can be detected and identified. In 2009, Tanimola et al. [14] applied DAS in intrusion threat recognition. Recent advancements in DAS signal processing have enabled vibration event detection and classification through deep learning architectures such as convolutional neural networks (CNNs) [15,16], long short-term memory (LSTM) networks [17], and hybrid models such as CLDNN [18], demonstrating superior performance in distinguishing pipeline intrusions and mechanical disturbances.

Following the identification of vibration events, accurately locating their sources is essential for assessing pipeline integrity. Hussels et al. [19] employed DAS to track propagating acoustic waves along the pipe wall and identify different acoustic modes, achieving transient impact localization. Huang et al. [20] proposed a pipeline inspection gauge (PIG) position method, which detects the vibration signals generated by the collision between PIG and pipeline welds, integrating a clustering algorithm and Hough transform. In addition, time-domain [21] and frequency-domain [22] cross-correlation algorithms are also used for the localization of pipeline vibration events.

However, the application of DAS in impact localization in large-diameter water pipelines remains challenging, mainly because of the difficulties in sensing fiber deployment. Existing research often utilizes an external deployment approach, typically burying the fiber alongside the pipe [14,15,16,17,18,20,22], which is suitable for new installations or shallow-buried pipelines. Another approach involves wrapping the fiber around the pipeline [19,21]. However, these approaches are impractical for existing, deeply buried water pipelines due to the high cost and restrictions of excavation. An alternative is to deploy the fiber internally within the pipeline. For instance, Higgins [23] and Lisbel [24] laid the fiber inside the pipeline without any fixation to detect wire breaks in PCCPs. However, this internal deployment approach is susceptible to background noise introduced by water flow. This noise field exhibits strong spatiotemporal variability along the fiber, rendering fixed filters ineffective. Consequently, transient impact signals, which are the target of this study, can be easily obscured, presenting a major challenge in accurate impact source localization.

The aforementioned studies on DAS signal processing and localization have not been validated under the noisy conditions associated with internal deployment in operational water pipelines. Although many algorithms have been developed for noise suppression in underwater environments, their inherent limitations render them unsuitable for the specific challenges encountered in this study. For instance, wavelet threshold denoising [25] requires prior knowledge to select the appropriate wavelet basis function and decomposition level, which becomes impractical in scenarios involving water flow noise. Furthermore, the least mean square algorithm [26] necessitates a reference noise signal, which is often unavailable when noise and target signals are inherently coupled. Another widely used method, Empirical Mode Decomposition [27], while capable of decomposing signals into intrinsic mode functions (IMFs), is known to suffer from mode-mixing, particularly when processing signals with transient features. Variational Mode Decomposition (VMD) [28] is an adaptive signal processing method designed to decompose a given signal into a predefined number of IMFs. Its principal advantage is the effective suppression of mode mixing, attributed to its non-recursive structure and narrow-band filtering mechanism. By decomposing a noisy signal into a set of modes, VMD effectively separates the signal components of interest from those primarily containing noise. In pipeline leakage detection, the optimized VMD approach effectively denoises acoustic signals contaminated by noise, enhancing feature extraction for further analysis [29].

The arrival time is typically defined as the point at which a noticeable difference emerges between noise and effective signal. The Autoregressive-Akaike Information Criterion (AR-AIC) algorithm [30] is a commonly used method for selecting the arrival time of seismic signals. It determines the optimal division point between noise and the effective signal at the minimum value of the AIC function. However, this method is limited to determining a single segmentation point for a given time series, whereas the actual arrival time may correspond to a local minimum of the AIC value. Pruned Exact Linear Time (PELT) [31] is able to precisely identify and segment all potential target vibration signals within long time series. Nonetheless, a challenge persists in that the unequal lengths of time series segments produced by PELT reduce the effectiveness of conventional metrics, such as Euclidean distance, when comparing features across different sequences.

Therefore, this paper proposes a novel two-step method designed for robust impact localization using a DAS-recorded signal from internally deployed, unfixed cables amidst strong water flow noise. The first step employs the VMD algorithm combined with a stable impulsiveness metric, Short-Time Energy Entropy (STEE), to adaptively extract the impact signal component from the noise. Subsequently, the second step achieves accurate automated selection of arrival time by applying the PELT algorithm, followed by an unsupervised learning method that uses Dynamic Time Warping (DTW) and clustering to identify the impact’s onset based on shape similarity, overcoming the limitations of traditional pickers in complex scenarios. The practical errors of this integrated approach are validated through field experiments on an operational water pipeline.

## 2. Sensing Principle of the DAS System

**Figure 1:**
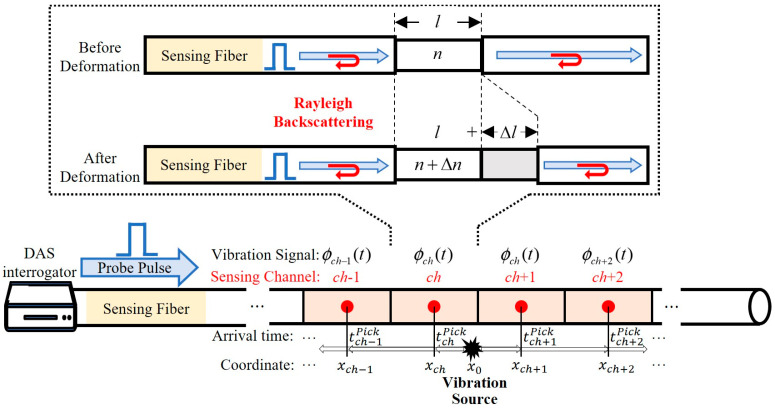
Schematic of the DAS principle.

The DAS system is based on phase-sensitive optical time-domain reflectometry, which mainly consists of an interrogator unit and an optical sensing fiber. Its measurement principle is shown in Figure 1. The DAS interrogator injects a probe laser pulse into the fiber. During the forward-propagation of the pulse, Rayleigh backscattering (RBS) signals are generated at different positions with different round-trip times. Deformation of the sensing fiber caused by external vibration leads to a phase change in the RBS signal, enabling the DAS system to measure dynamic strain changes along the fiber [13].

Within a segment of sensing fiber with a length of *l*, the phase delay of the return lightwave *ϕ* before deformation is as follows:

(1) $$\phi =2\beta n_{ref}l$$

where *β* is the wave vector in vacuum and *n_ref_* is the fiber refractive index.

After deformation is applied to this segment of fiber, the length and refractive index of the fiber change to Δ*l* and Δ*n_ref_*, respectively. The corresponding phase change can be expressed as follows [32]:

(2) $$\Delta \phi (x,t)=2\beta l\cdot \frac{\Delta l}{l}+2\beta l\cdot \Delta n_{ref}=2\beta (n_{ref}+C_{\varepsilon })l\cdot \varepsilon (x,t)$$

where *x* is the different location of the fiber, *t* is the temporal information, *ε*(*x*, *t*) = Δ*l*/*l* is the dynamic strain of the fiber, *C_ε_* = Δ*n_ref_*/*ε* is the constant coefficient of variation in the refractive index with strain, and *l* can be regarded as the spatial resolution of the DAS system, indicating the ability to separate the RBS in different positions. Therefore, the DAS system receives a vibration signal at every spatial resolution length, thereby converting the entire length of the fiber into a distributed sensing channel.

Since the purpose of this study is to achieve a linear positioning of the impact signal along the longitudinal axis of the pipeline, we used the time of arrival method, which was previously adopted by Hong for thunder source location [33]. Compared with point-type sensors, the key advantage of this approach lies in the DAS ability, ensuring high spatial density during vibration sampling. When a sufficiently strong impact occurs, numerous vibration arrival times can be selected within its wavefield range using DAS. This abundance of data reduces localization errors. As shown in Figure 1, after impact vibration occurs, stress waves, which are instantaneously generated, act on the fiber. Given the assumption that the stress waves propagate at a uniform speed *v* from the vibration source along the pipeline in the upstream and downstream directions, each sensing channel sequentially receives the vibration signal, in order from the closest to the farthest. The optimal vibration source location *x*_0_ can be obtained by minimizing the misfit between the calculated and chosen travel time:

(3) $$\begin{array}{l} \;\;\;t_{ch}^{cal}=\left|x_{ch}-x_{0}\right|/v\\ x_{0},t_{0}=\underset{x_{0},t_{0}}{\arg\min}\sum_{ch\in CH^{sel}}\left|t_{ch}^{cal}-(t_{ch}^{Pick}-t_{0})\right| \end{array}$$

where $t_{ch}^{cal}$ is the calculated travel time at channel *ch*, *x_ch_* is the coordinate of channel *ch* along the pipeline, $t_{ch}^{Pick}$ is the selected arrival time of the vibration signal at channel *ch*, *t*_0_ is the calculated initial time of vibration, and *CH^sel^* is the selected sensing channels in the wavefield’s range of vibration.

## 3. Method

### 3.1. Basic Process

In this study, we proposed a two-step method for locating impact signals within noisy DAS-recorded signals. The first step focuses on adaptively extracting the impact signal component from water flow noise. This is achieved via using VMD to decompose the raw signal into various IMFs, followed by applying STEE to quantify the impulsiveness of these IMFs and identify the specific IMF containing the primary impact vibration.

**Figure 2:**
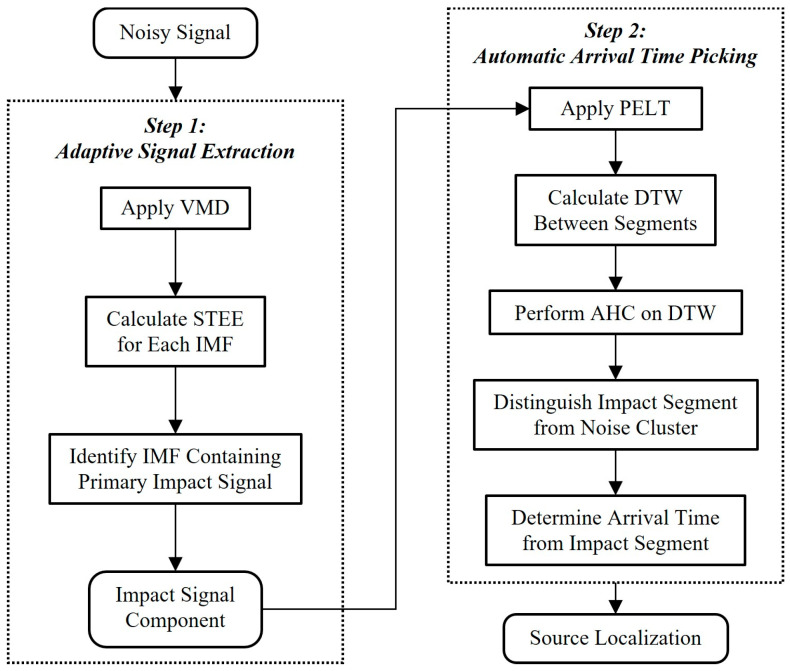
Flowchart of the proposed localization method.

Once the relevant impact signal component is selected, the second step automatically selects its arrival time. The PELT algorithm accurately segments the extracted signal by detecting all significant change points. Next, DTW measures the shape similarity between these variable-length segments. Finally, an unsupervised clustering method uses these DTW distances to group the segments, distinguishing the impact signal segment(s) from noise clusters based on structural dissimilarities, allowing the precise onset time to be determined. This process yields accurate arrival times, which are essential for source localization. The flowchart of the proposed method is shown in Figure 2. The proposed method was developed with Python 3.9.

### 3.2. Step 1: Adaptive Extraction of Impact Signals from Water Flow Noise Based on VMD-STEE

In this study, the VMD algorithm is employed to process signals recorded by DAS, enabling the extraction of impact-induced vibration signals from background water flow noise.

The main purpose of VMD is to identify several modes that are band-limited around a specific central frequency, ensuring that the summation of these individual modes accurately reconstructs the original input signal. This decomposition is achieved by formulating and solving a variational problem, where the primary objective is to minimize the sum of the bandwidths of all extracted modes. When the vibration signal *ϕ*(*t*) is decomposed into *K* modes, the objective function of VMD can be formulated as follows:

(4) $$\begin{array}{c} \underset{\left\{u_{k}\},\{\omega_{k}\right\}}{\min}\left\{\sum_{k=1}^{K}{\left\|\partial_{t}\left[\left(\delta (t)+\frac{j}{\pi t}\right)*u_{k}(t)\right]e^{-j\omega_{k}t}\right\|}_{2}^{2}\right\}\\ s.t.\;\sum_{k=1}^{K}u_{k}(t)=\phi (t) \end{array}$$

where $u_{k}\left(t\right)$ denotes the *k*-th mode decomposed from *ϕ*(*t*), $\omega_{k}\left(t\right)$ denotes the center frequency of the *k*-th mode, $\delta \left(t\right)$ is the Dirac delta function, j is an imaginary unit, the asterisk ∗ signifies the convolution operation, and $\partial_{t}$ represents the partial derivative with respect to time.

By incorporating the quadratic penalty factor *α* and a Lagrange multiplier *λ*(*t*), the constrained variational problem in Equation (4) is transformed into an unconstrained variational problem. The resulting augmented Lagrangian function expression is

(5) $$\begin{array}{c} L(\{u_{k}\},\{\omega_{k}\},\lambda )=\alpha \sum_{k}{\left\|\partial_{t}\left[\left(\delta (t)+\frac{j}{\pi t}\right)*u_{k}(t)\right]e^{-j\omega_{k}t}\right\|}_{2}^{2}\\ +{\left\|\phi (t)-\sum_{k}u_{k}(t)\right\|}_{2}^{2}+\langle \lambda (t),\phi (t)-\sum_{k}u_{k}(t)\rangle \end{array}$$

The saddle point of this augmented Lagrangian function in Equation (5) is then found using the Alternating Direction Method of Multipliers (ADMM). ADMM is an iterative optimization technique that updates the modes $u_{k}$, their corresponding center frequencies $\omega_{k}$, and the Lagrange multiplier *λ* in the frequency domain until a convergence criterion of relative tolerance and absolute tolerance is satisfied. In this study, the penalty factor was set as 2500, and the relative tolerance and absolute tolerance were set as 5 × 10^−3^ and 5 × 10^−6^, respectively.

Pre-setting an appropriate value for *K* is essential because this directly determines the quality of IMFs. Specifying a small *K* value leads to under-decomposition, where water flow noise contaminates the IMFs containing the impact signal. Conversely, a large *K* value results in over-decomposition, splitting the impact signal across multiple IMFs. Both scenarios impede the accurate selection of vibration arrival times in the next step. Various generalized information entropy measures, such as spectral entropy [34] and frequency band entropy [35], are widely applied across different fields, often serving to quantify specific characteristics of the time series data. Since the impact signal with transient characteristics is the object signal, this study uses the concept of STEE [36] to quantify the impulsiveness of IMFs derived from VMD. STEE is further utilized to adaptively determine the optimal value of *K* for VMD. Short-time energy reflects the temporal variations in signal energy. STEE is derived by calculating the information entropy of the energy distribution across all short-time windows within the signal segment. Its specific definition is provided below.

For the *k*-th IMF divided into *N* windows of length *w*, the short-time energy *E_k,n_* in the *n*-th window is

(6) $$E_{k,n}=\sum_{t=(n-1)*w+1}^{n*w}{\left[u_{k}(t)\right]}^{2}$$

STEE is then computed as

(7) $$\begin{array}{c} H_{k}=-\left[\sum_{n=1}^{N}P_{k,n}\ln(P_{k,n})\right]/\ln(N)\\ P_{k,n}=E_{k,n}/\sum_{i=1}^{N}E_{k,i} \end{array}$$

where *H_k_* represents the STEE of the *k*-th IMF, *N* is the total number of short-time windows, and *P_k,n_* denotes the proportion of energy in the *n*-th window relative to the total energy across all windows for the *k*-th IMF. Based on this definition, the STEE value is bounded between 0 and 1. Using STEE, the IMFs obtained from VMD can be classified into noise components and effective vibration components. A higher degree of transient behavior in an IMF, concentrated within a few short-time windows, results in a lower STEE value. Conversely, for a perfectly stationary signal where energy is evenly distributed (*P_k,n_* = 1/*N* for all *n*), the STEE value approaches its maximum of 1.

**Figure 3:**
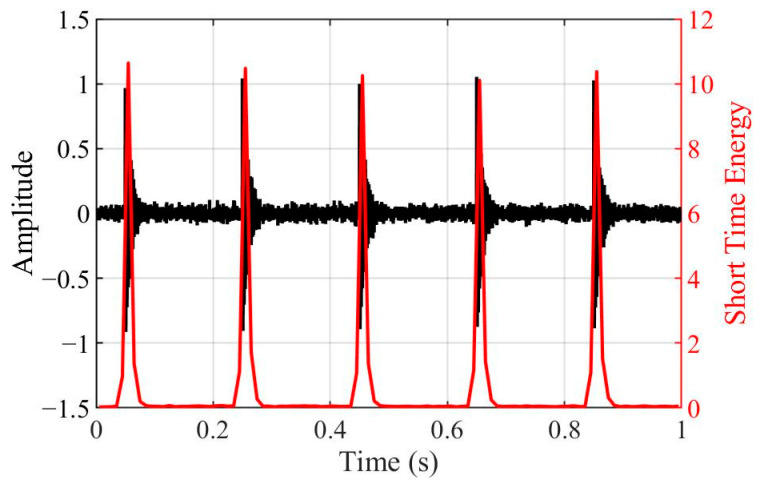
Simulated waveforms with five instances of impact signals and the corresponding STE sequences.

**Figure 4:**
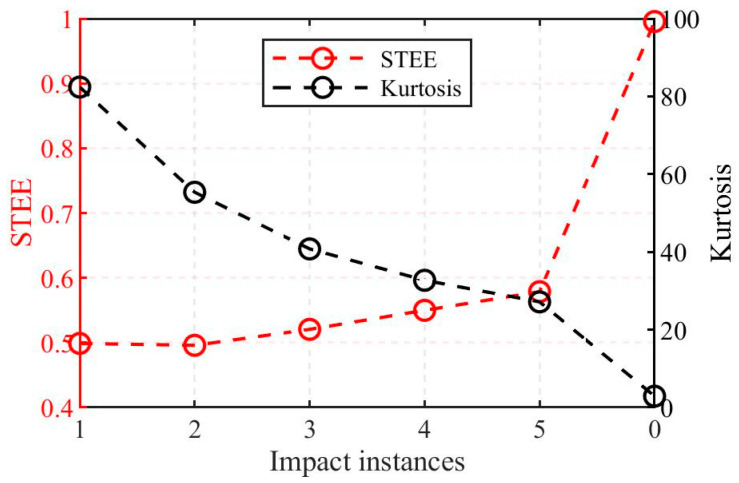
Comparison of kurtosis and STEE with different impact instances.

Kurtosis is a classical metric used to characterize the impulsiveness of a signal. However, its stability as an assessment tool can be limited in complex scenarios involving multiple impacts. In contrast, the proposed STEE exhibits lower sensitivity to the number of impacts, rendering it potentially more suitable as a stability indicator in such multi-impact scenarios. To illustrate this, consider a transient signal *f_T_*(*t*) was modeled as a damped sine wave, formulated as follows:

(8) $$f_{T}(t)=e^{-\kappa t}\sin(2\pi f_{c}t)$$

where *κ* is the time constant and *f_c_* is the central frequency of signal, set to 100 and 500 Hz, respectively. Figure 3 displays simulated waveforms within a 1 s window containing five instances of this impact signal. The corresponding Short-Time Energy (STE) sequences are plotted alongside this. To simulate ambient noise, white Gaussian noise with a power of −30 dBW was added to the signal. The STE sequences clearly show the concentration of signal energy around the impact events, exhibiting distinct peaks at these instants. Furthermore, Figure 4 presents the calculated kurtosis and STEE values for different impact instances. The results reveal a monotonic decrease in kurtosis as the number of impacts increases from one to five (from 82.39 to 27.15). In contrast, the STEE value shows only a modest increase (from 0.499 to 0.579). The baseline signal with zero impacts (representing background noise) exhibits the lowest kurtosis (2.90) and the highest STEE (0.995), approaching 1, as theoretically expected. The STEE values span a considerably smaller range compared to the kurtosis, which suggests that STEE offers superior stability when evaluating signals containing multiple transient impact events.

To effectively apply VMD to extract relevant vibration signals from water flow data, this study employs a stepwise incremental approach to determine the optimal number of *K* modes. The process starts with a minimum *K* of 2, which iteratively increases. At each step, the STEE is calculated for all resulting IMFs. If the minimum STEE value among all IMFs is less than a predefined threshold, the IMF corresponding to this minimum STEE is identified as the most prominent vibration component. This selected IMF is then utilized for subsequent arrival time determination. To prevent the potential misidentification of valid components caused by signal over-decomposition, the maximum value for *K* is set as 14. Furthermore, it is noted that the characterization of signal impulsiveness by STEE is influenced by the choice of short-time window length *w*. A detailed analysis of this parameter will be presented in the following sections.

### 3.3. Step 2: Automatic Arrival Time Selection Based on PELT and DTW-AHC

The PELT method builds upon the optimal partitioning method [37] by introducing a pruning strategy, allowing for the detection of change points with low computational cost and high accuracy. Its core idea is to find a segmentation that ensures the homogeneity of the statistical properties within each segment while maximizing the distinction between adjacent segments. Specifically, for a time series $y_{1:s}=(y_{1},\ldots ,y_{s})$ (representing the effective vibration signal), the set of segmentation points $T_{s}=\{\tau \;:0=\tau_{0}<\tau_{1}<\cdots <\tau_{M}<\tau_{M+1}=s\}$ is determined by minimizing the sum of fitting costs $F\left(s\right)$ within all segments, as shown in the following equation:

(9) $$\begin{array}{ll} F(s) & =\underset{\tau \in T_{s}}{\min}\left\{\sum_{i=1}^{M+1}\left[C(y_{(\tau_{i-1}+1):\tau_{i}})+\gamma \right]\right\}\\ & =\underset{\tau_{M}}{\min}\left\{\underset{\tau \in T_{M}}{\min}\sum_{i=1}^{M}\left[C(y_{(\tau_{i-1}+1):\tau_{i}})+\gamma \right]+C(y_{(\tau_{M}+1):s})+\gamma \right\}\\ & =\underset{\tau_{M}}{\min}\left\{F(\tau_{M})+C(y_{(\tau_{M}+1):s})+\gamma \right\} \end{array}$$

where C(·) represents the cost function for a segment, and *γ* is a penalty term for the number of change points. The equation recursively determines the last change point, *τ_M_*, based on the optimal partitioning up to that point. To improve computational efficiency, candidate change points *τ* that provably cannot be part of the optimal solution are pruned during each recursive step. Following the AR-AIC, the vibration signal is modeled with an assumption of normal variance, leading to a cost function defined as twice the negative log-likelihood.

Following segmentation by PELT, the DTW algorithm [38] is utilized to quantify shape similarity between the resulting segments with varying lengths. DTW finds the optimal alignment between two time series by non-linearly warping the time axis, thereby minimizing the cost required to match their shapes. Given two segments obtained by PELT, $y^{\left(P\right)}=(y_{1}^{\left(P\right)},\ldots ,y_{m}^{\left(P\right)})$ and $y^{\left(Q\right)}=(y_{1}^{\left(Q\right)},\ldots ,y_{n}^{\left(Q\right)})$, a warping path $WP=\{{wp}_{1},\ldots ,{wp}_{L}\}$ defines the alignment between their corresponding elements. The goal of DTW, subject to monotonicity constraints, is to find the path that minimizes the cumulative distance $DTW(y^{\left(P\right)},y^{\left(Q\right)})$ over all possible paths, expressed as follows:

(10) $$\begin{array}{c} DTW(y^{\left(P\right)},y^{\left(Q\right)})=\underset{WP}{\min}\left[\sum_{i=1}^{L}d(wp_{i})\right]/L\\ d(p,q)=\left\|y_{p}^{\left(P\right)}-y_{q}^{\left(Q\right)}\right\| \end{array}$$

where *L* is the length of the warping path. Normalizing by *L* accounts for the fact that longer signals tend to accumulate larger total distances, providing the distance per unit path length. d(p,q) signifies the Euclidean distance between elements $y_{p}^{\left(P\right)}$ and $y_{q}^{\left(Q\right)}$. The optimal warping path is found recursively by backtracking through the distance matrix from the final cell (aligning the full sequences) to the initial cell. The cumulative distance ∆(p,q) for each cell is calculated as follows:

(11) Δ(p,q)=d(p,q)+min[Δ(p−1,q),Δ(p−1,q−1),Δ(p,q−1)]

This formula indicates that the cumulative distance ∆(p,q) is the sum of the distance d(p,q) at the current cell and the minimum cumulative distance from the valid neighboring cells to (p,q). The DTW distance between the two sequences is the value of the final element of the cumulative distance matrix, ∆, signifying the minimum cost to align them.

After calculating the pairwise distances between all PELT-generated segments using DTW, these distances are used for clustering.

For impact signal segment detection, this study employs an unsupervised learning framework that combines DTW with a clustering method. The onset time of the detected impact segment is determined to be the arrival time. Based on the DTW distance matrix, a hierarchical clustering algorithm [39] is applied to structure the time segment data, which can be visualized as a dendrogram. This procedure operates on the assumption that noise segments tend to cluster together at lower distance thresholds, forming a large main cluster. In contrast, signal segments, due to their distinctiveness, remain separate and merge with the main noise cluster only at a much larger distance. Consequently, the significant separation distance between the impact signal cluster and the noise cluster can be used as an indicator to distinguish them; for instance, the maximum inter-cluster merge distance can be used for this purpose. This unsupervised approach avoids the need to preset the number of clusters and directly leverages the structural dissimilarity within the data to identify the impact signal. Finally, the arrival time of the signal can be selected automatically.

## 4. Field Test

**Figure 5:**
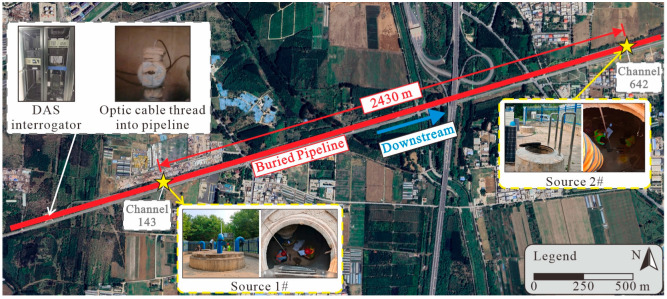
Map of the experiment site. The thick red line depicts the buried pipeline. The yellow stars mark impact Sources 1# and 2#, located 2430 m apart along the pipeline.

In this study, an impact experiment was conducted on an in-service water pipeline to verify the proposed localization method, as shown in Figure 5. The pipeline mainly consists of PCCPs. It features an inner diameter of 4 m and comprises individual sections of 5 m in length. The pipelines are connected via the spigot and the bell method. Its burial depth varies from 3 m to 9 m. During the experiment, the water flow rate within the pipeline was maintained at 21 m^3^/s. This corresponds to an average internal flow velocity of approximately 1.67 m/s, which is close to the upper limit of the economical flow velocity range of from 0.9 to 1.8 m/s [40]. The tested pipeline section is designed for a maximum flow rate of 25 m^3^/s, so the experimental flow condition reflects a typical operating condition for this pipeline.

The DAS system is manufactured by Ningbo AllianStream Photonics Technology Co., Ltd., Ningbo, China. The type of DAS interrogator used in the test was ixDAS-2000, which recorded signals along the fiber with a spatial resolution of 4.91 m and a sampling frequency of 5000 Hz. The system operated with a pulse width of 50 ns, and the acquisition card had a sampling frequency of 250 MHz. The refractive index of the optical fiber was 1.467. The sensing optic cable, encasing the fiber, was deployed longitudinally inside the pipeline without being fixed, and mainly recorded the vibration signals propagating through the water medium. It is a custom cable, suitable for use in drinking water and designed with strong waterproofing and mechanical strength to withstand the internal pipeline environment. The cable was threaded through a special cable entry assembly installed on the steel pipe section, and laid in the downstream direction.

To simulate potential TPI events, impacts were manually induced on the pipeline using two types of rebound hammers with different impact energy (IE) levels: a concrete rebound hammer (IE = 2.2 J) and a high-strength concrete rebound hammer (IE = 4.5 J). The rebound hammers are manufactured by Beijing Hichance Technology Co., Ltd., Beijing, China. The varying impact energy levels provided by the two hammer types were intended to simulate different threat scenarios, such as manual excavation or mechanical drilling activities near the pipeline. Prior to the experiment, the rebound hammers were calibrated to ensure consistent IE delivery. Impact positions were selected at two accessible air valve chambers (Source 1# and Source 2#), as indicated in Figure 5. These two impact locations were situated 2430 m apart along the pipeline axis. At each location, impacts were induced 10 times using each type of hammer. The location results from the two distinct impact points allowed for cross-validation of the method’s errors against the known ground truth positions of the impacts.

## 5. Results

### 5.1. Characteristics of Noisy Impact Signal

**Figure 6:**
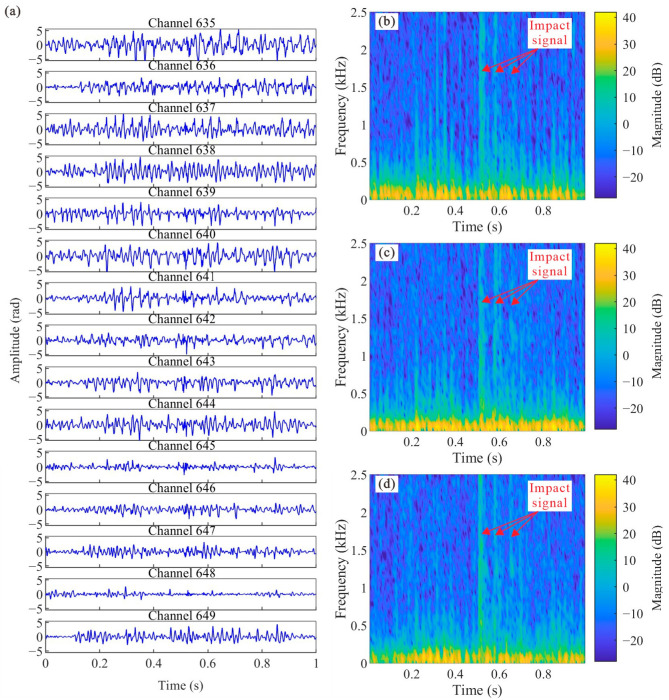
(**a**) Noisy impact signal (IE = 2.2 J) recorded by DAS system from channel 635 to channel 649 at Source 2#. Corresponding spectrogram based on STFT for (**b**) channel 639, (**c**) channel 640, and (**d**) channel 641, respectively.

In this study monitoring impact signals within an in-service pipeline using an internally deployed, unfixed optic cables presented significant challenges due to the noise environments. Figure 6 shows a representative 1 s segment of raw data recorded by the DAS system during an impact event (IE = 2.2 J) induced at Source 2#. Figure 6a displays the noisy vibration waveforms across adjacent sensing channels (from 635 to 649), while Figure 6b–d provide the corresponding spectrograms using the magnitude of the Short-Time Fourier Transform (STFT) for channels 639, 640, and 641, respectively, located near the impact.

The time-domain waveforms in Figure 6a show substantial spatiotemporal variability in the background noise, despite the relatively stable water flow rates during the experiment. For example, channels 635–640 exhibit persistently high noise levels, whereas the noise amplitude on channel 648 is significantly lower than its neighbor channels. However, none of the time-domain signals exhibited clear characteristics indicative of the impact event.

The spectrograms in Figure 6b–d demonstrate the signal composition. Continuous vibrations associated with background water flow disturbances predominantly manifested in the low-frequency range (below approximately 500 Hz), an observation consistent with previous findings from accelerometer-based pipeline monitoring [6]. The impact event, appearing between approximately 0.5 s and 0.6 s, is characterized by broadband energy distributed across a wide frequency range, which appears as distinct, vertically oriented bands in the spectrograms, reflecting its impulsive nature. During impact, the rebound hammer’s striker rod experienced successive collisions with the pipeline wall, manifesting in the spectrogram as three tightly spaced vertical bands with a progressively diminishing intensity, analogous to acoustic reverberations. The near-simultaneous appearance of these features across adjacent channels underscores the high spatial sampling density afforded by DAS. Notably, the spectrogram for channel 639 (Figure 6b) exhibits a localized surge of high-frequency components between 0.2 s and 0.4 s, extending beyond the typical flow noise band. This phenomenon is indicative of flow-excited noise, likely resulting from cable motion or oscillations induced by unstable local flow conditions.

**Figure 7:**
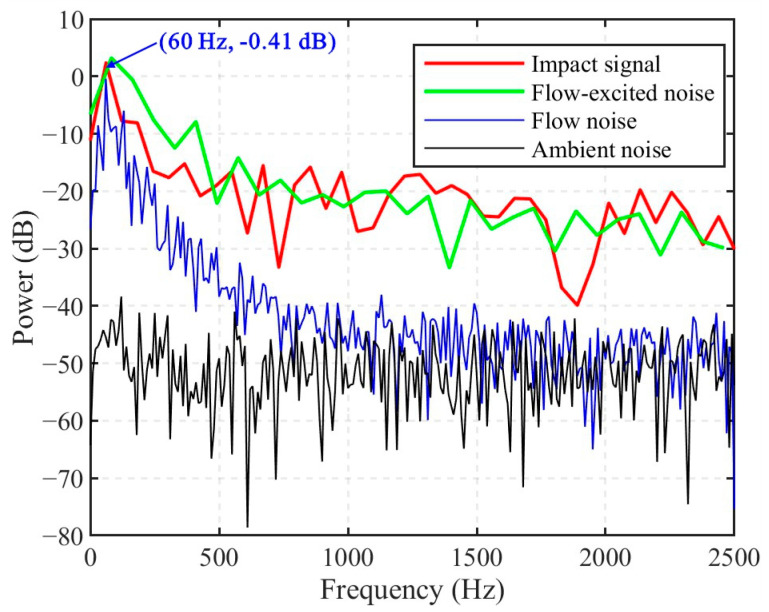
Power spectra of different signals. The impact signal is obtained from 0.5154 s to 0.5316 s of channel 639 in Figure 6a. The flow-excited noise corresponds to 0.3110–0.3230 s, and the flow noise to the first 0.1 s of the same channel. The ambient noise is obtained from signals recorded outside the pipeline, not contaminated by flow noise.

Furthermore, Figure 7 presents the power spectra of different types of DAS-recorded signals. The ambient noise exhibits the flattest power distribution and the lowest overall power, mostly ranging from −70 dB to −40 dB. In contrast, the flow noise shows significantly higher power than ambient noise below 500 Hz, with a peak at 60 Hz corresponding to a power of −0.41 dB. Since both the impact signal and the flow-excited noise contain components of the flow noise, they exhibit a similar peak frequency and corresponding power levels. A notable feature of both the impact signal and the flow-excited noise is their higher power in the frequency range above 500 Hz, typically between −30 dB and –20 dB. However, distinguishing between these two signals in this frequency band remains challenging due to their spectral overlap.

These characteristics—the significant spatiotemporal variability and low-frequency dominance of the noise, the broadband nature of the impact signal, and potential artifacts from cable dynamics—show the complexity of the recorded signals. Therefore, accurately identifying the impact arrival time directly from the raw time-domain waveforms is challenging due to the presence of complex background noise. This necessitates the adoption of an adaptive signal processing approach to effectively extract the impact signal.

### 5.2. Signal Extraction Based on VMD-STEE

To evaluate the performance of STEE in characterizing the impulsiveness of impact signals and determine the optimal analysis window length, *w*, this study investigated STEE’s effect on classifying signal components. The dataset comprised 240 impact signals (derived from 40 impact events, selecting six adjacent channels per event near the source) and 240 time slots of pure water flow noise. All signals were decomposed into six IMFs using VMD. The minimum STEE value among the IMFs was calculated for each signal sample using different window lengths *w*, which were set to 50, 100, 150, 200, 250, and 300. This minimum STEE served as the classification feature for subsequent analyses.

**Figure 8:**
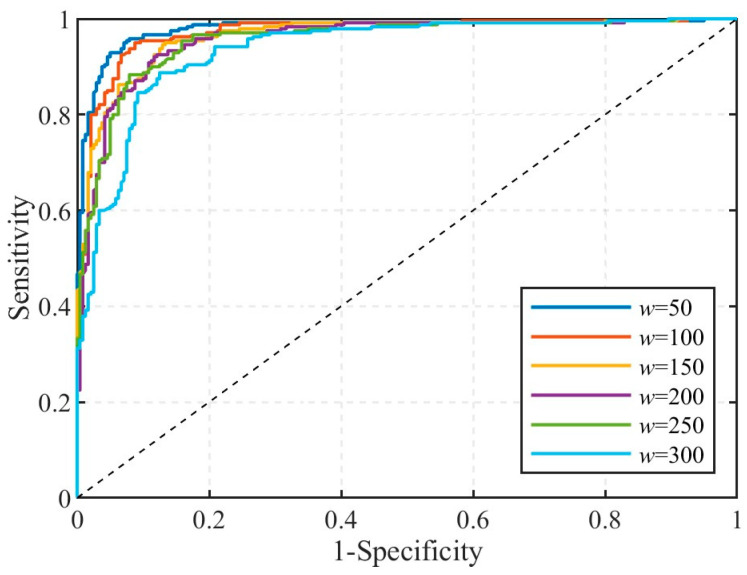
ROC curve with different STEE window length.

Receiver Operating Characteristic (ROC) analysis was used to assess the discriminatory capability of the minimum STEE feature. An ROC curve is a graphical representation plotting the True Positive Rate against the False Positive Rate at various classification thresholds. The Area Under the Curve (AUC) provides a single metric summarizing the overall ability of the classifier to distinguish between positive (impact) and negative (noise) classes across all thresholds, with values closer to 1 indicating better performance. An optimal threshold is typically selected from the ROC curve, the point closest to the ideal top-left corner, to balance sensitivity and specificity for practical classification. Figure 8 displays the ROC curves generated for the different STEE window lengths *w*. The calculated AUC values (presented in Table 1) quantify this overall performance. The results show high AUC values for most window lengths, confirming the strong discriminatory power of the minimum STEE feature. Notably, *w* = 50 yielded the highest AUC (0.979), suggesting that this provides the best overall separation between impact and noise signals across all possible thresholds. A slight decreasing trend in AUC was observed as *w* increased, with a more significant drop at *w* = 300 (AUC = 0.936).

To evaluate the performance at the optimal threshold, several specific metrics were calculated:

1. Precision: The proportion of instances predicted as positive (impact signals) that are truly positive. High precision indicates few false alarms among the identified impacts.
2. Specificity: The proportion of actual negative instances (noise signals) that are correctly identified as negative. High specificity means the classifier effectively identifies noise.
3. Sensitivity: The proportion of actual positive instances (impact signals) that are correctly identified as positive. High sensitivity means the classifier detects most of the true impact signals.
4. Accuracy: The overall proportion of correct classifications (both positive and negative) among all instances. This measures the overall correctness of the classification.

Table 1 presents these performance metrics, which were calculated at the optimal STEE threshold determined for each window length *w*. The analysis of these specific metrics reveals that *w* = 50 not only had the best overall AUC but also achieved the highest precision (94.87%) and specificity (95.00%) at its optimal threshold.

The choice of window lengths is primarily driven by the requirement to avoid misclassifying background noise as impact signals (requiring high specificity) while also correctly identifying the true impact signals (requiring high precision). The results in Table 1 clearly indicate that *w* = 50 satisfies these requirements at the optimal threshold, thereby maximizing the reliability of impact signal identification for the subsequent STEE calculations and mitigating the potential adverse effects of noise misclassification on the final localization errors.

**Figure 9:**
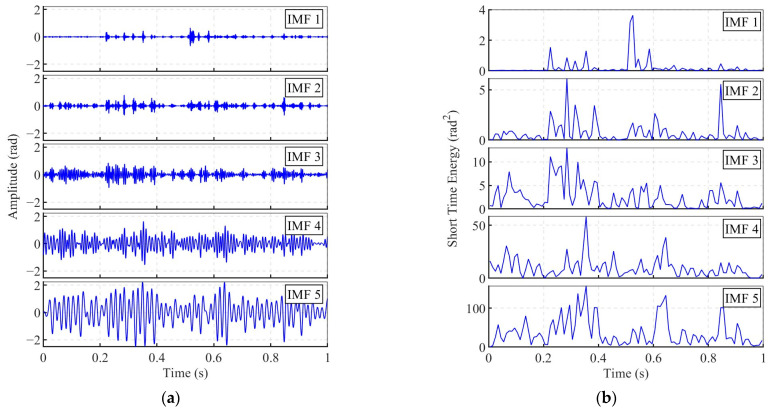
(**a**) Decomposed IMFs of the noisy impact signal and (**b**) the corresponding STE results.

Figure 9 shows the decomposition results of the channel 639 signal (from Figure 6a) based on the proposed VMD-STEE method, including the IMFs and their corresponding STE sequences. The STEE values and central frequencies of the decomposed components are summarized in Table 2. As demonstrated in the results, only IMF1 exhibits distinct transient characteristics. The peak in STE temporally aligns with the vertical impulse bands observed in the spectrogram of Figure 6b. IMF1 features the lowest STEE value (0.704), reflecting its concentrated energy distribution during transient impacts, and a central frequency of 1006.0 Hz, consistent with the broadband nature of impact-induced vibrations. In contrast, the STE sequences of IMF2–IMF5 lack pronounced energy peaks, and their STEE values exceed 0.84, making them characteristic of background noise or quasi-stationary signal components rather than transient impacts. Notably, the impact arrival time is considerably more discernible in IMF1 compared to the raw signal in Figure 6a. IMF5 exhibits a relatively stable waveform, with the highest energy amplitude and a low central frequency (56.2 Hz), suggesting that it corresponds to the hydrodynamic noise caused by water flow disturbances and their dynamic interactions with the sensing cable. Notably, this central frequency closely matches the peak frequency of the water flow noise observed in Figure 7, further supporting this interpretation.

### 5.3. Arrival Time Selection Based on PELT and DTW-AHC

**Figure 10:**
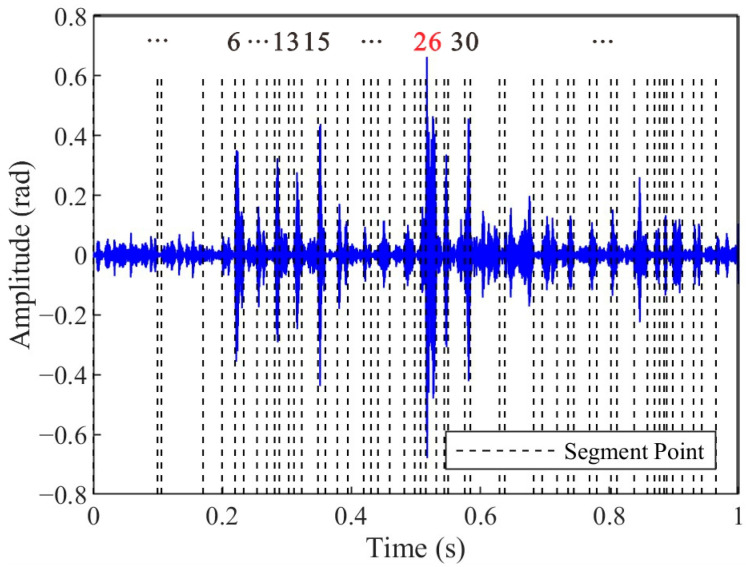
Segmentation results of the extracted impact signal based on the PELT method. The impact segment is denoted as Segment 26 (in red).

Figure 10 shows the segmentation results of the impact signal extracted from Figure 9a IMF1 based on the PELT method. The results demonstrate that the original impact signal is partitioned into 53 segments, where the change points correspond to abrupt transitions in signal characteristics. Segments 26 and 30 exhibit distinct transient features that temporally align with the impact event, as evidenced by their sharp amplitude transitions and spectral coherence with the broadband characteristics of impact-induced vibrations, which can be seen in Figure 6b. In contrast, segments 6, 13, 15, as well as others, also exhibit high amplitudes, but they lack temporal and spectral coherence with the impact signals. These results show that the PELT algorithm provides a precise segmentation basis for a subsequent similarity-based clustering analysis.

**Figure 11:**
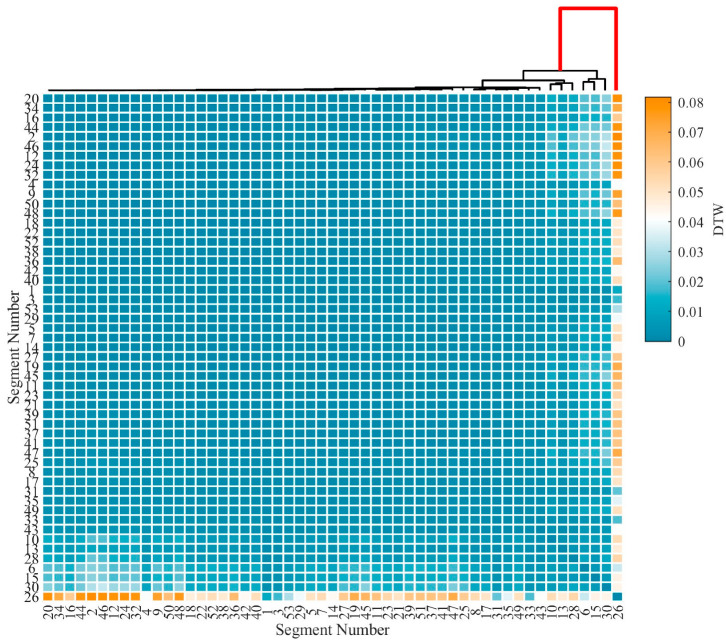
Arrival time selection of the extracted impact signal based on DTW-AHC. The red line represents the maximum inter-cluster merge distance.

Figure 11 presents the arrival time selection results based on the DTW-AHC method when applied to the segments shown in Figure 10, visualized as a clustering heatmap. The DTW distances between segments were calculated using Equation (10), followed by the construction of a hierarchical clustering dendrogram via average linkage. In the heatmap, color intensity reflects the shape similarity between segments, with darker hues indicating higher similarity. The clustering results show that noise segments, such as background water flow disturbances, form a dominant cluster at low merge distances. In contrast, impact signal segments, characterized by their distinct morphological features, only merge with the main cluster at higher merge distances. By analyzing the maximum inter-cluster merge distance threshold, which is indicated by the red line in the dendrogram at Segment 26, the onset time of the impact signal can be accurately determined.

**Figure 12:**
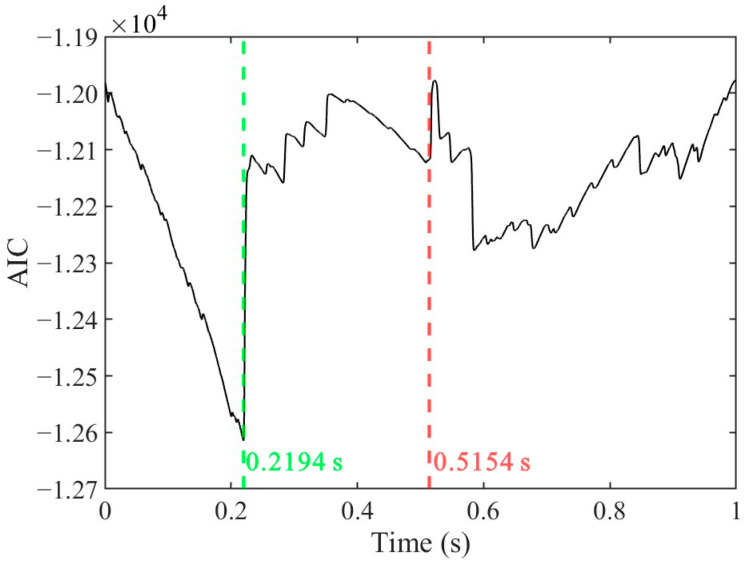
The AIC sequence of the extracted impact signal. The red dashed line is the arrival time determined based on the method proposed in this paper, and the green dashed line is the arrival time determined based on the AR-AIC method.

Figure 12 illustrates the AIC sequence of the extracted impact signal in Figure 10, comparing the arrival time determination results of the proposed method and the AR-AIC. The proposed method identifies the onset time of the impact signal at 0.5154 s, corresponding to a local minimum in the AIC sequence. However, the AR-AIC method selects the global minimum at 0.2194 s, which corresponds to the start time of segment 6. This discrepancy arises because the AR-AIC method, being limited to the detection of a single segmentation point, fails to distinguish between transient signal onsets and transient noise in scenarios with multiple change points. In contrast, the proposed PELT-DTW-AHC framework comprehensively segments the signal and evaluates segment similarity, enabling the precise identification of the initial transient feature, without being unaffected by the length of the time window.

**Figure 13:**
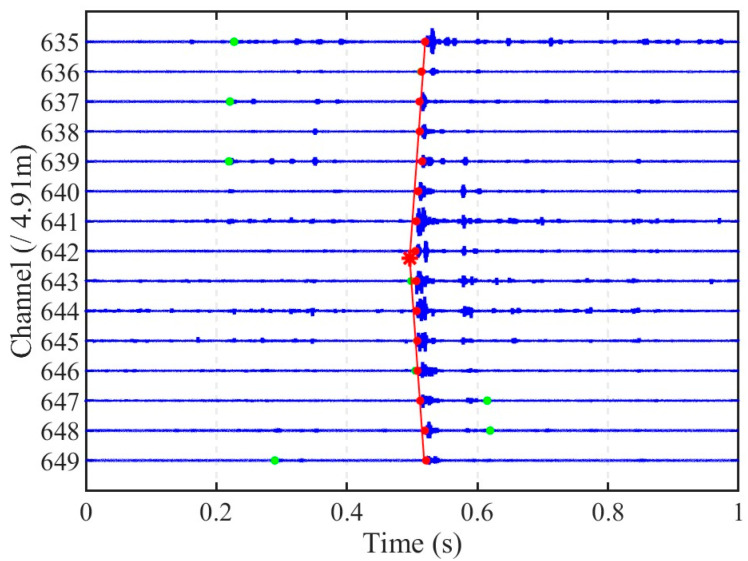
Arrival time selection from channels 635 to 649. The red dots indicate the arrival time determined based on the method proposed in this paper, the red line shows the corresponding minimized misfit function, and the red asterisk highlights the source location. The green dots show the arrival time determined using the AR-AIC method.

Figure 13 presents the arrival time selection results for the impact event shown in Figure 6 across channels 635 to 649. The red line illustrates the minimized misfit function derived from Equation (3), where the wave propagation velocity *v* is set to 1190 m/s, calculated based on the theoretical propagation speed of pressure waves in an elastic pipe [41]. The optimized localization result, positioned 1.18 m downstream of channel 642, is marked by a red asterisk in Figure 13. Specifically, the AR-AIC method failed to select the correct arrival times in channels 635, 637, 639, 647, 648, and 649, often selecting earlier or later points that are inconsistent with the overall propagation pattern. In contrast, the proposed approach exhibited more consistency in arrival time identification across channels.

### 5.4. Source Location

**Figure 14:**
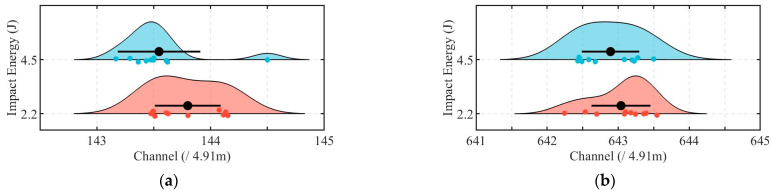
Statistical results of impact location at (**a**) Source 1# and (**b**) Source 2#, with different impact energy levels. The results are illustrated using half-violin plots, with the mean values and standard deviations represented by black dots and lines.

The field impact experiments conducted in this study were designed not only to validate the localization errors of the proposed method, but also to confirm the spatial division of the DAS system sensing channels within the pipeline, a prerequisite for its practical application. Figure 14 presents the statistical results of impact localization for two distinct impact sources (Source 1# and Source 2#) under varying impact energy levels (IE = 2.2 J and 4.5 J). At Source 1#, the standard deviations of localization results were 1.42 m and 1.78 m for IE = 2.2 J and IE = 4.5 J, respectively, with corresponding ranges (maximum difference between calculated locations) of 3.32 m and 6.55 m. Similarly, at Source 2#, the standard deviations measured 1.95 m and 1.98 m for the two energy levels, while the ranges were 5.96 m and 5.25 m, respectively. The outlier observed in Figure 14a, corresponding to an impact with IE = 4.5 J, was caused by strong noise interference, which disrupted the arrival time selection and led to a deviated localization between Channels 144 and 145. These results indicate consistent localization performance across different impact energy levels and positions using the proposed method. Notably, the average calculated source positions across all impact events were 3.30 m downstream of channel 143 for Source 1#, and 4.76 m downstream of channel 642 for Source 2#, corresponding to an optic cable separation of 2451.55 m. However, the actual pipeline distance between the two impact locations was 2430 m, yielding a cumulative positioning error of 21.55 m. This discrepancy arises from two factors: (1) the inherent limitations of the proposed localization method under practical field conditions, and (2) unavoidable slack in the sensing cable installation within the pipeline to prevent mechanical failure caused by its self-weight, resulting in a slightly longer cable length compared to the pipeline. However, the normalized error per meter of pipeline is approximately 0.009 m (21.55 m/2430 m), which is negligible for practical applications.

**Figure 15:**
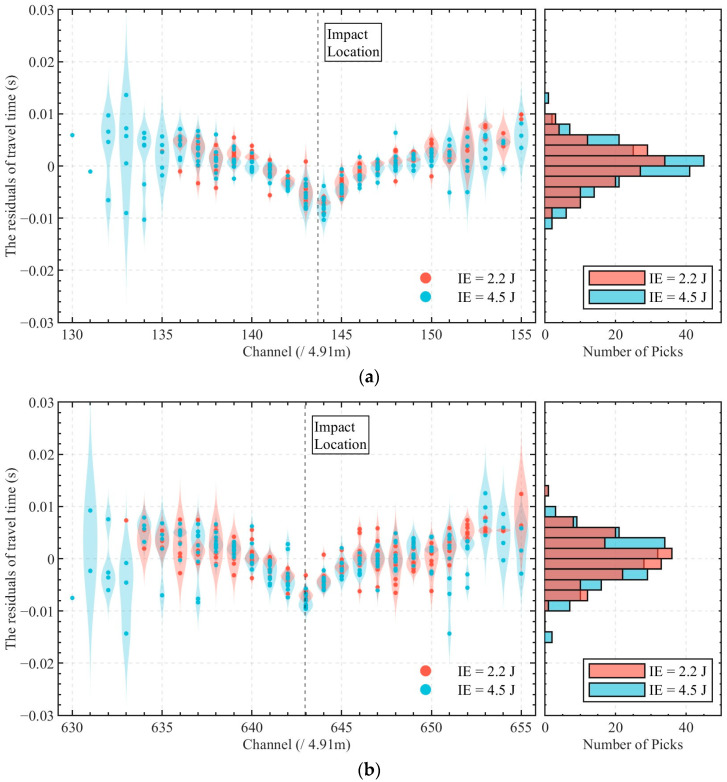
Distribution of travel time residuals of all impact events at (**a**) Source 1# and (**b**) Source 2#, respectively. The left panel displays the residual distribution for each channel, represented by scatter points and their normalized probability density estimations, while the right panel presents a statistical histogram of all residuals.

Figure 15 presents the statistical distribution of travel time residuals, defined as $t_{ch}^{cal}-(t_{ch}^{Pick}-t_{0})$, across all sensing channels for a total of 697 picked arrival times. These comprise 346 picked arrival times from impact events at Source 1# and 351 picked arrival times from Source 2#. Here, $t_{0}$ represents the optimal initial time calculated for each impact event, and $t_{ch}^{cal}$ denotes the theoretical travel time derived from the average source location $x_{0}$. The residuals predominantly cluster within the range of −0.1 s to 0.1 s, with a near-zero mean and standard deviations spanning 0.0038 s to 0.0045 s under different conditions, suggesting consistent performance in arrival time selection and the localization model. Channels located closer to the impact source exhibit lower dispersion in their residuals, indicating higher precision in arrival time selection in those regions. In contrast, distal channels exhibit broader residual spreads. For impacts with higher energy (IE = 4.5 J), the vibration signals propagate over longer distances, resulting in more arrival times being selected on channels farther from the source. However, these arrival times tend to show larger deviations from the calculated travel times, which contributes to the slightly larger overall dispersion observed in the localization results for the 4.5 J impacts, as shown in Figure 14. Notably, the residuals near the source are predominantly negative (indicating earlier calculated arrivals), while the residuals at distal channels skew positive (suggesting delayed calculated arrivals). This spatial pattern suggests non-uniform wave propagation dynamics. Near the source, vibrations propagate through both the pipeline wall and water medium, with faster solid-borne waves dominating initial arrivals. As the wavefront travels farther, solid-borne components attenuate, leaving slower fluid-borne waves as the primary signal detected by the cable. This transition explains the systematic shift in residuals and highlights the limitations in assuming a constant wave velocity across all propagation paths.

## 6. Discussion

A novel two-step method was presented for localizing impact vibration sources on pipelines using DAS-recorded data. The main challenge lies in accurately detecting and timing the arrival of transient impact signals, particularly in operational scenarios characterized by high spatiotemporal noise variability and overlapping transient signals, when using internally deployed, unfixed sensing cables.

In the first step, the proposed VMD-STEE method proved suitable for adaptively extracting the relevant impact signal component observed from the operational pipeline. As shown in Figure 6, the background noise exhibits significant spatiotemporal variability across different sensing channels. The use of STEE provided a metric for quantifying the impulsiveness of each mode, allowing it to be reliably distinguished from the more continuous flow-induced noise regardless of its specific amplitude or frequency content. ROC analysis obtained an optimal short-time window length for STEE calculation, maximizing the precision and specificity needed to reliably identify the impact component while minimizing false positives due to noise misclassification. As shown in Figure 9, IMF1—associated with the impact event and exhibiting the lowest STEE—is clearly distinguishable from background noise components such as IMF4. This distinction facilitates accurate arrival time determination for subsequent localization.

**Figure 16:**
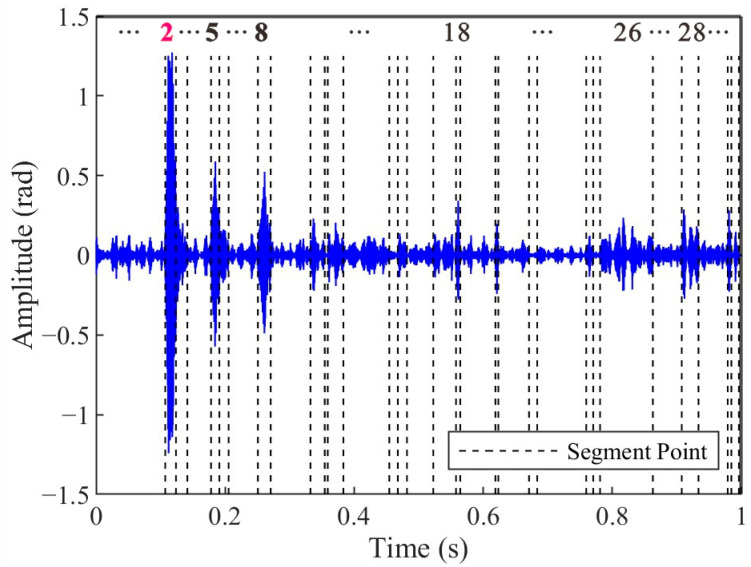
Segmentation results of the extracted impact signal recorded at Source 1#. The impact segment is denoted as Segment 2 (in red).

The second step, combining PELT and DTW-AHC, focused on accurate arrival time selection, segmenting signals into homogeneous regions and clustering them based on shape similarity. Under typical conditions, as shown in Figure 16, the amplitude of the selected impact signal segments (Segment 2) significantly exceeded that of the noise segments. Nevertheless, the arrival time selection method proposed in this study demonstrates robust performance even in complex-noise environments. During the experiment, the water flow rate was maintained at 21 m^3^/s, with a Reynolds number of 6.7 × 10^6^, indicating a fully turbulent flow regime. Under such hydrodynamic conditions, the optical cable experienced continuous oscillation due to water flow, leading to dynamic deformations in the optical fiber. As shown in Figure 10, despite the signal extraction by the VMD-STEE, considerable noise persisted in the extracted impact signal. Notably, Segments 6, 13, and 15 exhibited higher amplitudes compared to other segments. Despite this, the subsequent arrival time selection step, depicted in Figure 11, effectively identifies the true impact. Hierarchical clustering shows that Segment 26 displayed a markedly larger merging distance than other clusters, confirming it as the primary impact signal. These results validate that the proposed method captures the correct onset of impact signals, overcoming the limitations of threshold-dependent or global-optimization-based approaches in complex vibration environments.

Field tests under operational pipeline conditions validated the practical applicability of the proposed method. Localization results were consistent across two distinct source locations and two impact energy levels, achieving standard deviations typically between 1.42 m and 1.98 m. For repeated impacts at the same location, the maximum difference between calculated positions did not exceed 6.55 m, which is shorter than the length of the two pipe sections. These results provide a reliable foundation for structural assessments of pipelines.

The results also indicate the need for further investigation. While STEE demonstrated reliable performance in detecting manual impacts, its effectiveness in identifying low-energy vibrations has yet to be validated. The integration of machine learning classifiers trained on a variety of anomalous vibration patterns may extend the method’s applicability to broader scenarios. Additionally, as shown in Figure 15, the analysis of travel time residuals highlights the complex physics of wave interaction within the pipe structure and the fluid medium. The trend of negative residuals near the source and positive residuals farther away suggests a transition in the dominant wave-propagation path. This observation indicates the limitation of assuming a constant wave velocity for all propagation paths and distances, although the near-zero mean of the residuals suggests that this assumption provides a reasonable average fit.

## 7. Conclusions

This study proposed and validated a two-step method for localizing impact vibration sources on pipelines using DAS-recorded signals that were heavily contaminated by water flow noise. The first step, utilizing VMD-STEE, effectively extracts impact signal components by adaptively decomposing the noisy signal and leveraging the STEE metric to reliably identify the most impulsive IMF, demonstrating superior stability compared to kurtosis for signals with multiple impacts. The second step, combining PELT segmentation with DTW-AHC clustering, enables accurate automatic arrival time selection by identifying change points and clustering segments based on shape similarity, proving robust even when noise amplitudes rival impact signal amplitudes and outperforming traditional methods like AR-AIC in scenarios with multiple change points. Field experiments conducted on an in-service water pipeline validated the method’s practical applicability, achieving consistent localization errors with standard deviations between 1.42 m and 1.98 m under varying impact energies and locations. The proposed framework advances pipeline monitoring by enabling reliable damage localization in noisy environments, which is critical for infrastructure safety.

## Figures and Tables

**Table 1:** Classification performance and optimal threshold of STEE with different window lengths.

Window Length	AUC	Optimal Threshold	Precision	Specificity	Sensitivity	Accuracy
50	0.979	0.722	94.87%	95.00%	92.50%	93.75%
100	0.971	0.717	94.07%	94.17%	92.50%	93.33%
150	0.963	0.702	93.28%	93.33%	92.50%	92.92%
200	0.957	0.710	91.36%	91.25%	92.50%	91.88%
250	0.955	0.737	88.19%	87.50%	93.33%	90.42%
300	0.936	0.723	87.50%	86.67%	93.33%	90.00%

**Table 2:** The STEE and central frequency of different components decomposed by VMD.

Component	STEE	Central Frequency (Hz)
IMF1	0.704	1006.0
IMF2	0.843	367.1
IMF3	0.891	179.6
IMF4	0.920	104.8
IMF5	0.918	56.2

## Data Availability

The raw data supporting the conclusions of this article will be made available by the authors on request.
